# The interplay between oncolytic viruses, Toll-like receptor signaling, and chemotherapy in overcoming tumor immune tolerance

**DOI:** 10.3389/fcimb.2026.1898807

**Published:** 2026-07-31

**Authors:** Yi-Ying Wu, Feng-Hsu Wu, Chen-Yuan Tseng, Pin-Kuan Chiang, Muhammad Muniri, Hung-Jen Liu

**Affiliations:** 1 Institute of Molecular Biology, National Chung Hsing University, Taichung, Taiwan; 2 The iEGG and Animal Biotechnology Center, National Chung Hsing University, Taichung, Taiwan; 3 Division of General Surgery, Department of Surgery, Taichung Veterans General Hospital, Taichung, Taiwan; 4 Department of Critical Care, Taichung Veterans General Hospital, Taichung, Taiwan; 5 Department of Nursing, Hung Kuang University, Taichung, Taiwan; 6 Division of Biomedical and Life Sciences, Faculty of Health and Medicine, Lancaster University, Lancaster, United Kingdom; 7 Rong Hsing Research Center for Translational Medicine, National Chung Hsing University, Taichung, Taiwan; 8 Department of Life Sciences, National Chung Hsing University, Taichung, Taiwan

**Keywords:** chemotherapy, immune tolerance, immunogenic cell death, innate immunity, oncolytic viruses, Toll-like receptors, tumor microenvironment, type I interferon

## Abstract

Oncolytic viruses (OVs) represent a unique therapeutic platform that combines tumor-selective replication with potent immunomodulatory capacity. In addition to direct oncolysis, OVs can convert tumors into inflammatory niches that support antigen release, dendritic cell activation, and cytotoxic T lymphocyte priming. However, durable responses remain inconsistent across tumor types and patient populations, largely due to tumor immune tolerance mechanisms that restrict both viral propagation and the development of effective anti-tumor immunity. Toll-like receptors (TLRs), as key pattern recognition receptors, play central roles in sensing viral nucleic acids and infection-associated danger signals, orchestrating type I interferon responses, NF-κB-driven inflammation, and downstream adaptive immunity. Notably, TLR signaling is a double-edged sword in virotherapy: it can promote antigen presentation and immune activation while simultaneously accelerating antiviral clearance and limiting intratumoral viral spread. Chemotherapy, a mainstay of cancer treatment, further shapes OV efficacy by altering lymphocyte availability, antigen presentation, myeloid composition, and the balance between immunogenic cell death and immunosuppression. Emerging evidence supports that rational OV-chemotherapy combinations can synergize by enhancing tumor antigen release, reprogramming suppressive myeloid compartments, and creating temporal windows for immune checkpoint blockade. In this review, we summarize the mechanisms underlying tumor immune tolerance, discuss how TLR-mediated innate sensing shapes OV-induced anti-tumor immunity, and evaluate how chemotherapy modulates these interactions. We propose mechanistic frameworks and translational considerations including scheduling, biomarkers, and immune monitoring to guide the development of next-generation OV combination strategies in chemotherapy-treated cancer patients.

## 1 Introduction

Cancer progression is tightly coupled to immune escape and the establishment of immune tolerance within the tumor microenvironment (TME). Although tumors harbor neoantigens and stress-associated ligands that can be recognized by innate and adaptive immune cells, malignant lesions frequently evolve to evade detection. This immune tolerance is characterized by ineffective antigen presentation, expansion of regulatory populations, myeloid-driven suppression, and T-cell dysfunction (Tufail et al., 2025). Over the past decade, immune checkpoint blockade has demonstrated that reversing immune suppression can yield durable clinical benefit. Yet, checkpoint inhibitors alone are often insufficient for many solid tumors, particularly “cold” tumors characterized by low baseline T-cell infiltration or dominant myeloid-mediated immunosuppression. Therefore, novel therapeutic modalities that can simultaneously induce tumor cell death and ignite productive inflammation have become increasingly important (Adashek et al., 2025).

Oncolytic viruses (OVs) occupy a distinctive niche in this landscape, bridging virology, immunology, and oncology. Unlike conventional cytotoxic agents, OVs replicate selectively within malignant cells to induce immunogenic tumor destruction. In addition to direct oncolysis, OVs function as *in situ* vaccines by promoting tumor antigen release and providing pathogen-associated molecular patterns (PAMPs) that activate innate immunity, effectively converting immunologically cold tumors into hot tumors (Wang et al., 2023; Wu et al., 2023; Shi et al., 2024). These properties have fueled the development of multiple naturally occurring or genetically engineered OV platforms, including adenoviruses, herpes simplex viruses (HSV), vaccinia viruses, reoviruses, measles viruses, and vesicular stomatitis virus (VSV). From a clinical perspective, the approval of talimogene laherparepvec (T-VEC) established OV therapy as a validated anti-cancer strategy. However, response rates remain variable, and the majority of patients do not achieve durable remission with OV monotherapy (Bahreyni et al., 2024). A key reason for this limitation is that OV therapy must navigate two opposing immunological constraints. On one hand, successful anti-tumor immunity requires robust innate sensing and inflammatory programming that supports antigen presentation and T-cell priming. Conversely, these antiviral responses can lead to premature viral clearance, limiting intratumoral spread and diminishing oncolytic efficacy. This tension is especially evident in Toll-like receptor (TLR) signaling (Liu et al., 2020). TLRs detect viral nucleic acids and infection-associated danger signals, triggering interferon regulatory factor (IRF) activation, NF-κB signaling, and production of type I interferons (IFN-I) and inflammatory cytokines. TLR signaling is therefore essential for the immunogenicity of OV infection but can also restrict viral replication and therapeutic persistence (Kircheis and Planz, 2023).

Chemotherapy adds an additional layer of complexity. Many cancer patients receiving OV therapy have prior or concurrent exposure to chemotherapy, which can reshape immune competence, antigen presentation, and the TME (Merlano et al., 2022). While chemotherapy is traditionally considered immunosuppressive, it can also enhance anti-tumor immunity by inducing immunogenic cell death (ICD), depleting regulatory immune subsets, and increasing tumor antigen availability. Conversely, chemotherapy can drive lymphopenia, impair dendritic cell function, and promote myeloid rebound, which may reinforce immune tolerance. Importantly, chemotherapy can influence the timing and magnitude of antiviral and anti-tumor responses during OV therapy, making scheduling a critical determinant of clinical outcome (Phan et al., 2024). This review focuses on the mechanistic intersection of three core themes: tumor immune tolerance as a barrier to effective OV therapy, TLR-mediated innate sensing as a central regulator of OV-induced immunity, and chemotherapy as both a potentiator and antagonist of OV-based immunotherapy. We emphasize the conceptual framework that OV therapy is not merely a viral cytotoxic strategy but an immune reprogramming approach, whose success depends on the careful tuning of innate sensing pathways and the immunological context established by chemotherapy. Understanding these interactions is essential for designing rational combination regimens, identifying predictive biomarkers, and developing next-generation OVs optimized for durable tumor control.

## 2 Core biological framework and immunological foundations of oncolytic virus therapy

### 2.1 Conceptual foundations of tumor-selective replication and permissiveness

Oncolytic viruses preferentially infect tumor cells and exploit vulnerabilities in tumor antiviral defenses. Many malignant cells display impaired type I interferon (IFN-I) signaling, defects in dsRNA sensing, dysregulated cell cycle control, altered apoptosis pathways, and oncogenic pathway activation that collectively favor viral gene expression and unchecked replication (Xiao et al., 2026). For example, aberrant Ras/MAPK signaling can enhance replication of certain RNA viruses, while defective p53 pathways can facilitate replication of DNA viruses.

This concept of tumor permissiveness is central to OV selectivity, although some tumors retain strong antiviral responses and remain poorly permissive, contributing to heterogeneous clinical efficacy (Xiao et al., 2026). OV platforms can be broadly categorized as naturally tumor-selective viruses (e.g., reovirus, Newcastle disease virus, avian reovirus) or engineered viruses with tumor-specific attenuation and immunostimulatory payloads (e.g., HSV-1, adenovirus, vaccinia) (Jadhav et al., 2025). Genetic engineering strategies include deletion of viral genes required for replication in normal cells, insertion of tumor-selective promoters, and expression of cytokines or immune-modulating molecules such as GM-CSF, IL-12, chemokines, bispecific T cell engagers, or checkpoint inhibitors (Jadhav et al., 2025).

### 2.2 Direct oncolysis, cell death modalities, and tumor debulking

The most intuitive mechanism of OV therapy is direct oncolysis. Viral replication within permissive malignant cells culminates in host cell lysis, structural collapse, and local tumor debulking. However, clinical evidence suggests that direct lysis alone rarely accounts for durable systemic control (Xiao et al., 2026). In many models, long-term benefit requires adaptive immunity, and OV-induced tumor regression is attenuated in immunodeficient hosts. Therefore, oncolysis is best viewed as the initiating event that generates inflammatory tumor debris and antigenic material rather than the sole therapeutic driver (Xiao et al., 2026).

Crucially, this replication and subsequent destruction do not merely destroy the cell mechanically; viral replication drives a variety of regulated cell death pathways, inducing ICD, apoptosis, and autophagy, which further prime and amplify anti-tumor immunity (Dou et al., 2024).

### 2.3 Innate immune activation, TLR signaling cascades, and macrophage dynamics

OV infection triggers innate immune sensing within the TME through pattern recognition receptors (PRRs), including TLRs, RIG-I-like receptors, and cGAS-STING pathways. This multi-pathway activation leads to the robust production of type I IFNs, pro-inflammatory cytokines, and chemokines, promoting the subsequent recruitment and activation of dendritic cells (DCs), natural killer (NK) cells, and macrophages (Khan et al., 2026).

Within this innate cascade, TLR signaling plays a nuanced and pivotal role. While the distinct functional role of cell-surface versus intracellular TLR3 has been historically characterized in chronic inflammatory pathologies like proliferative inflammatory atrophy (PIA) (Chen et al., 2021), this spatial regulation offers critical insights into the TME. Specifically, this cell-surface localized TLR3 logic directly applies to how tumor-associated macrophages (TAMs) sense extracellular viral or dying-cell nucleic acids, a pivotal determinant in shifting macrophage polarization from a pro-tumorigenic M2 phenotype toward an antitumorigenic M1 state during oncolytic therapies. In the context of viral oncology, Avian reovirus (ARV) is increasingly recognized as an OV with the capacity to selectively infect and eliminate malignant cells while simultaneously engaging host innate immune responses. Accumulating evidence suggests that ARV infection activates TLR3-dependent signaling pathways in tumor cells, resulting in the downstream activation of interferon regulatory factor 3 (IRF3) and nuclear factor kappa B (NF-κB) (Cao et al., 2025).

Several studies have proposed that interactions between ARV structural proteins, particularly sigmaC, and cytoplasmic TLR3 contribute to the nuclear translocation of IRF3 and NF-κB and the subsequent induction of interferon-gamma (IFN-gamma). This IFN-gamma-mediated signaling engages the Janus kinase-signal transducer and activator of transcription 1 (JAK-STAT1) pathway, which plays a central role in coordinating antiviral responses, autophagy, and programmed cell death. Together, these observations support a model in which ARV-driven activation of the TLR3-IRF3/NF-κB/IFN-gamma/JAK-STAT1 signaling network directly links innate immune recognition to autophagy-associated oncolytic effects (Hsu et al., 2025).

### 2.4 Immunogenic cell death and *in situ* vaccination

A central immunological feature of effective OV therapy is the formal induction of ICD. As the tumor cell undergoes oncolysis, it releases a potent combination of tumor-associated antigens (TAAs), viral PAMPs, and damage-associated molecular patterns (DAMPs). This ICD is specifically characterized by the emission of key DAMPs, such as surface-exposed calreticulin, ATP release, HMGB1 release, and the local production of IFN-I and other inflammatory cytokines (Fayyad-Kazan et al., 2026).

These danger signals recruit and mature antigen-presenting cells (APCs), particularly dendritic cells (DCs). The activated DCs process the released TAAs and neoantigens, migrating to secondary lymphoid organs to cross-present these tumor antigens to CD8^+^ cytotoxic T lymphocytes and CD4^+^ helper T cells (Wu et al., 2023, 2024, 2025a, c).

OV infection can thus transform a cold tumor lesion into a highly inflammatory *in situ* vaccine, driving a systemic and durable anti-tumor immune response. This includes the establishment of immunological memory capable of controlling distant metastatic disease and preventing recurrence. Importantly, this process broadens the repertoire of T cell responses through epitope spreading, contributing to long-term therapeutic control even after the virus itself has been cleared from the host (Giram et al., 2025).

### 2.5 Remodeling the tumor microenvironment and overcoming stromal barriers

Beyond initiating cell death, OVs comprehensively remodel the cellular and structural architecture of the TME. Upon viral infection, the subsequent up-regulation of chemokines such as CXCL9, CXCL10, and CCL5 orchestrates the direct trafficking and infiltration of effector T cells and NK cells into the tumor core. OV therapy can also increase the expression of endothelial adhesion molecules and modify tumor vasculature, significantly improving immune cell infiltration. Conversely, this highly inflammatory microenvironment often drives the compensatory upregulation of immune checkpoints, including PD-L1, establishing a clear mechanistic foundation for synergistic combinations with checkpoint inhibitors (Park et al., 2026).

Simultaneously, the physical stromal architecture of solid tumors represents a major barrier to effective therapy. Dense extracellular matrix (ECM), high interstitial fluid pressure, and abnormal vasculature can severely limit viral delivery, penetration, and intratumoral dissemination. To overcome these barriers, strategies such as carrier cell delivery, regional or intratumoral administration, and OVs engineered to express ECM-degrading enzymes (e.g., hyaluronidase) have been explored, though managing the associated systemic safety considerations remains an ongoing translational focus (Choi et al., 2013).

### 2.6 Immune dominance and the balance between antiviral clearance and anti-tumor immunity

A defining translational challenge in OV therapy is that host antiviral immunity directly competes with anti-tumor immunity. Pre-existing neutralizing antibodies (NAbs), complement activation, and rapid innate immune clearance can restrict viral persistence, spread, and secondary infections within the tumor mass. While these clearance processes act as a natural safety check to prevent systemic toxicity, they also limit intratumoral replication and diminish overall therapeutic potency (Martinez-Quintanilla et al., 2019).

Conversely, excessive dampening of the host’s antiviral immunity can raise clinical safety risks and may paradoxically reduce the initial immune sensing necessary for robust immune priming. Therefore, modern OV design and combination strategies aim to optimize a precise therapeutic window: one in which viral replication is sustained long enough to induce robust immunogenic tumor destruction, while host innate sensing is tuned to support optimal antigen presentation and the induction of systemic adaptive immunity (Panetti et al., 2022).

## 3 Tumor immune tolerance as cellular and molecular barriers to oncolytic virotherapy

**Figure 1:**
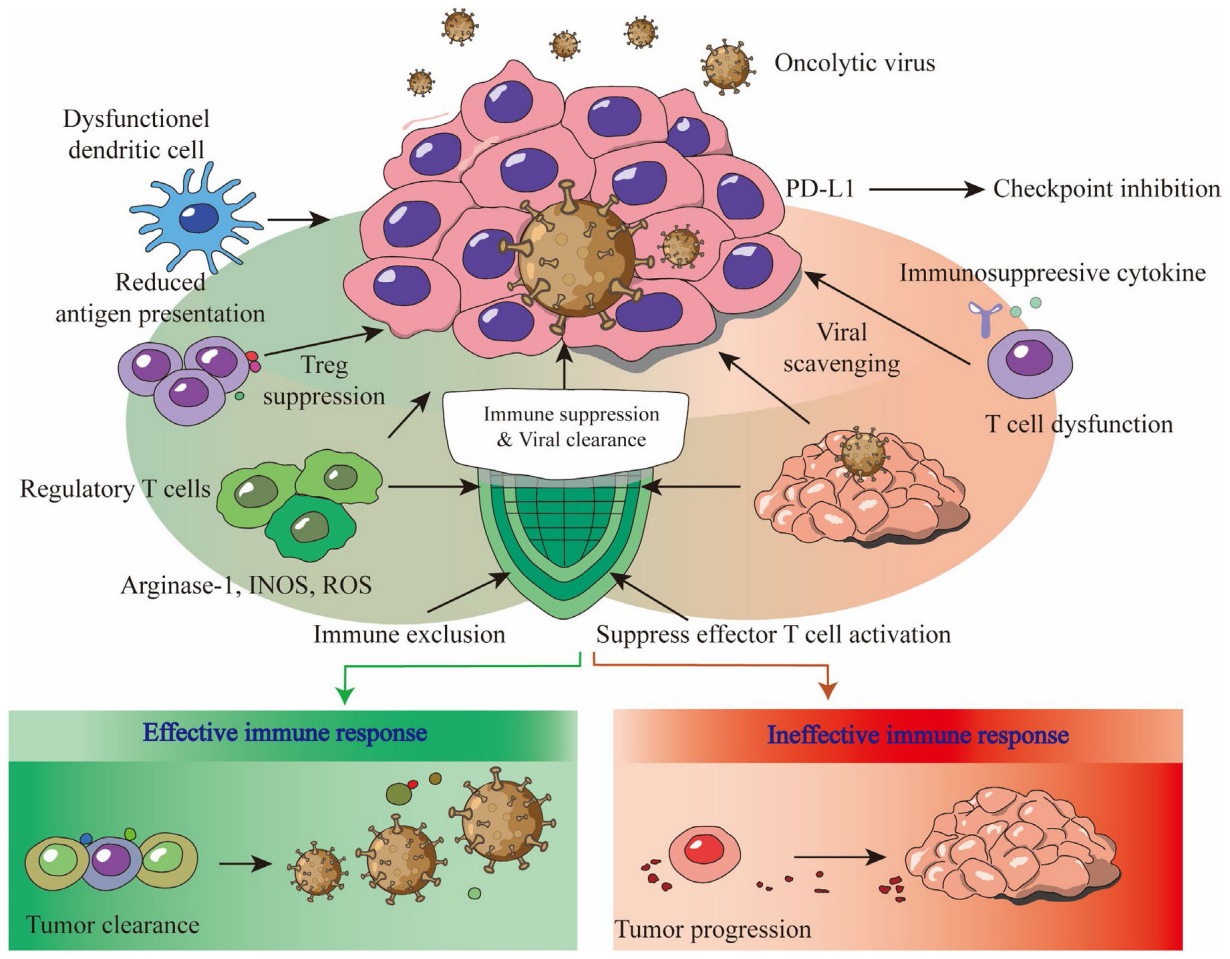
Tumor immune tolerance mechanisms shaping oncolytic virotherapy outcomes. Schematic illustrating the major immune tolerance pathways that limit OV efficacy. Tumor cells and stromal components suppress antigen presentation, promote inhibitory cytokines (IL-10 and TGF-β), and upregulate checkpoint ligands (PD-L1). Regulatory T cells suppress effector T cell activation and support immune exclusion. Myeloid-derived suppressor cells and tumor-associated macrophages restrict T cell function through arginase-1, iNOS, ROS, and immunosuppressive cytokines while also scavenging viral particles and limiting intratumoral spread. Dendritic cell dysfunction reduces cross-presentation of tumor antigens released during OV-mediated immunogenic cell death. These barriers collectively determine whether OV infection induces productive immune priming and systemic anti-tumor immunity or results in transient inflammation followed by viral clearance and tumor immune escape.

Tumor immune tolerance is best understood as an emergent state generated by multiple reinforcing mechanisms rather than a single dominant pathway. For OV therapy, immune tolerance is particularly consequential because it suppresses both arms required for durable efficacy: intratumoral immune activation needed for *in situ* vaccination and effector responses required for systemic tumor control. Furthermore, tumors employ various strategies to evade immune surveillance-including antigen loss, downregulation of major histocompatibility complex (MHC) molecules, and the secretion of immunosuppressive cytokines such as TGF-β and IL-10 (Ostrand-Rosenberg, 2016; Wu et al., 2023, 2025a). Beyond immune evasion, tolerance indirectly limits OV spread by promoting myeloid-mediated viral scavenging and maintaining stromal barriers that restrict intratumoral dissemination (**Figure 1**).

### 3.1 Antigen presentation defects and dendritic cell dysfunction

Efficient anti-tumor immunity requires the cross-presentation of tumor antigens by professional APCs, particularly conventional dendritic cells (cDC1), which are specialized for priming CD8^+^ T cells. Many tumors suppress this axis through strategies including the downregulation of antigen processing and presentation machinery, reduction of MHC class I expression, and the exclusion of dendritic cells from the tumor core. Even when OVs trigger immunogenic cell death, the antigenic material may fail to generate productive priming if DC maturation is impaired or if the TME blocks DC trafficking (Fu and Jiang, 2018).

OVs can partially overcome DC dysfunction by providing PAMPs and inflammatory cytokines that drive DC activation. However, tumors frequently impose a dominant suppressive tone that blunts DC licensing. This is clinically relevant because patients with low baseline DC signatures often show reduced response to immunotherapies, and OV-induced inflammation may not be sufficient to reverse this deficit without additional interventions (Chen et al., 2024).

### 3.2 Regulatory T cells and tolerance reinforcement

Regulatory T cells (Tregs) represent a central tolerance mechanism in many solid tumors. They suppress effector T cell proliferation and cytokine production through pathways including IL-10/TGF-β secretion, CTLA-4-dependent suppression of APC co-stimulation, and metabolic competition for IL-2. Tregs also contribute to immune exclusion by shaping chemokine networks and supporting a suppressive stromal niche (Chaudhary and Elkord, 2016).

In OV therapy, Tregs exert a dual influence. While OV infection can promote inflammatory cytokines that transiently reduce Treg dominance and enhance effector infiltration, antiviral inflammation may also induce compensatory Treg expansion as a host-protective mechanism to limit immunopathology. This dynamic partially explains why OV therapy often induces strong early inflammation but fails to maintain long-term effector function (Fayyad-Kazan et al., 2026).

### 3.3 Myeloid-derived suppressor cells and tumor-associated macrophages

Among tolerance mechanisms, myeloid populations-specifically myeloid-derived suppressor cells (MDSCs) and tumor-associated macrophages (TAMs)-are often dominant in shaping OV outcomes. MDSCs suppress T cell activation via arginase-1-mediated arginine depletion, inducible nitric oxide synthase (iNOS), reactive oxygen species (ROS), and immunosuppressive cytokines. TAMs, particularly those polarized toward an M2-like state, support angiogenesis, tissue remodeling, and immune suppression through the production of IL-10, TGF-β, and the expression of checkpoint ligands (Dash et al., 2026).

For OV therapy, these populations impose constraints beyond T cell suppression. TAMs and MDSCs act as innate antiviral barriers by phagocytosing viral particles, producing antiviral cytokines, and limiting intratumoral spread. Thus, a myeloid-dominant TME can simultaneously limit viral persistence and suppress the adaptive immune responses required for systemic antitumor efficacy (Wu et al., 2023). This dual barrier has driven increasing interest in myeloid reprogramming as a key strategy in the development of next-generation OV-based therapies (Wu et al., 2024).

### 3.4 Immune checkpoint pathways and T cell exhaustion

Chronic antigen exposure and persistent inhibitory signaling contribute significantly to T cell dysfunction and exhaustion. This state is characterized by reduced effector function and sustained expression of inhibitory receptors such as PD-1, CTLA-4, TIM-3, LAG-3, TIGIT, and VISTA (Wu et al., 2023, 2025a, c).

OV therapy often induces IFN-driven upregulation of PD-L1 on tumor and myeloid cells, which can be beneficial when combined with PD-1/PD-L1 blockade but may limit efficacy in monotherapy settings. Importantly, OV infection can transiently increase T cell infiltration without necessarily restoring durable cytotoxicity. In such cases, the TME shifts from an immune-excluded state to an inflamed-but-suppressed phenotype-representing the precise clinical scenario where combining checkpoint blockade with myeloid-targeting therapies becomes essential to secure a sustained therapeutic benefit (El-Sayes et al., 2022).

### 3.5 Stromal exclusion, vascular abnormalities, and physical barriers

Solid tumors frequently exhibit abnormal vasculature, hypoxia, and dense extracellular matrix (ECM), which limit immune infiltration and OV dissemination. Cancer-associated fibroblasts (CAFs) contribute to ECM deposition and recruit suppressive myeloid cells. High interstitial pressure reduces convection and diffusion, impairing the delivery of both immune cells and viral particles (Belhabib et al., 2021).

Consequently, the OV delivery method is a critical variable. Intratumoral injection bypasses some systemic barriers but is not feasible for all lesions. Intravenous delivery frequently encounters neutralizing antibodies, complement activation, and hepatic or splenic sequestration. To overcome these systemic barriers, researchers are exploring strategies such as polymer shielding, regional delivery, and the use of cell carriers like mesenchymal stem cells (Deeson et al., 2026).

## 4 Toll-like receptor signaling in oncolytic virotherapy: molecular mechanisms and host immunological responses

**Figure 2:**
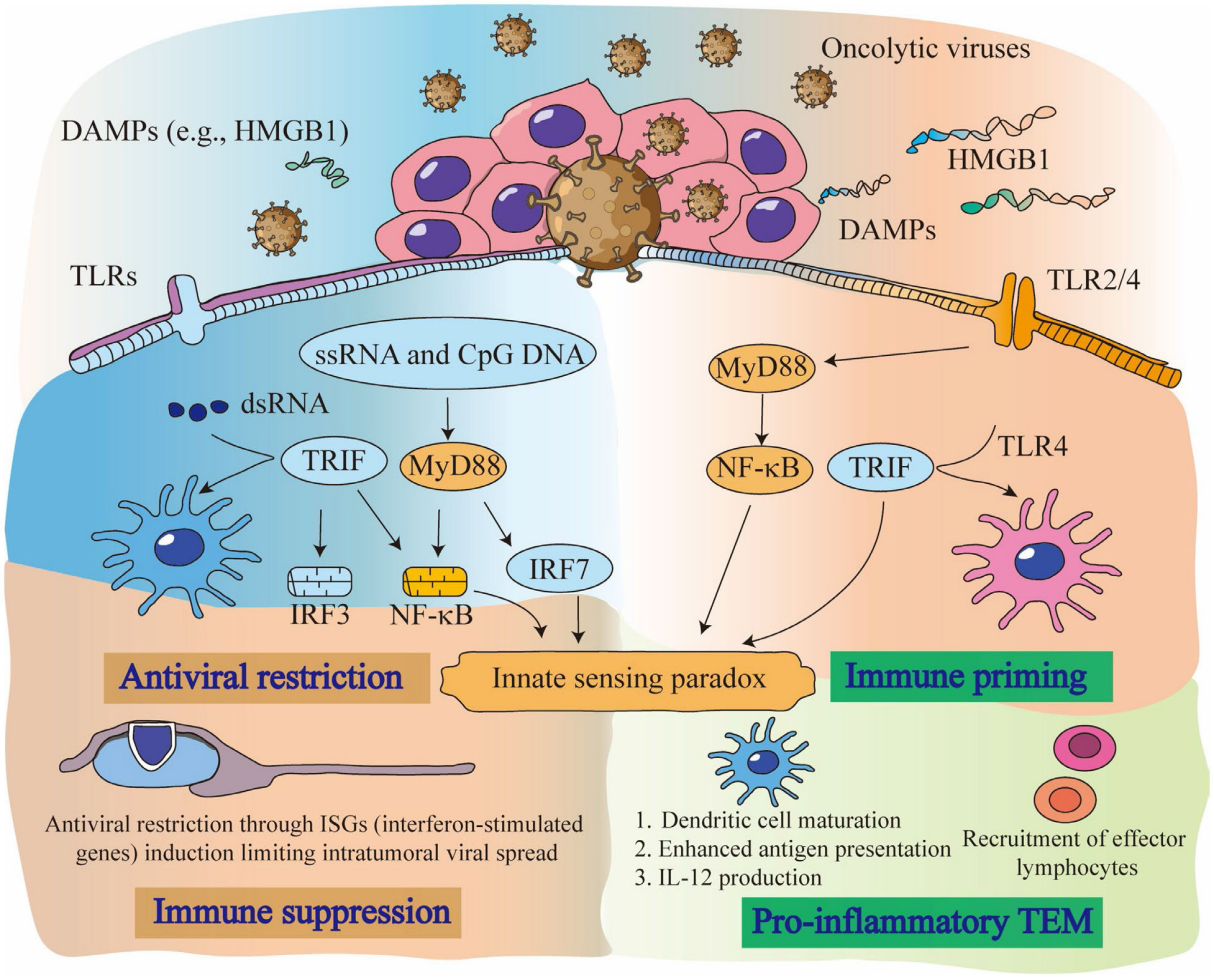
Toll-like receptor signaling pathways shaping oncolytic virotherapy outcomes. Schematic overview of TLR pathways engaged during oncolytic virus infection and tumor cell death. Within the endosomal compartment, TLR3 recognizes dsRNA and utilizes the TRIF adapter protein to activate IRF3 and NF-κB, which subsequently induces the expression of type I interferons (IFN-I) and pro-inflammatory cytokines. In contrast, endosomal TLR7/8, which senses ssRNA, and TLR9, which detects CpG DNA, propagate signals predominantly through the MyD88-dependent pathway; this cascade activates NF-κB and IRF7 to drive the production of both inflammatory cytokines and IFN-I in a cell-type–specific manner. Surface TLR2 and TLR4 detect viral proteins and DAMPs (e.g., HMGB1), leading to MyD88-dependent NF-κB activation; TLR4 can additionally engage TRIF. Downstream outputs include antiviral restriction through ISG induction limiting intratumoral viral spread and immune priming through dendritic cell maturation, enhanced antigen presentation, IL-12 production, and recruitment of effector lymphocytes. The image highlights the innate sensing paradox, where TLR-driven antiviral responses can restrict OV replication while being required for effective anti-tumor immunity.

The clinical integration of oncolytic virotherapy relies heavily on TLR signaling, which dictates whether infection precipitates productive anti-tumor adaptive immunity or triggers premature antiviral restriction. As OVs function simultaneously as cytotoxic agents and viral adjuvants, TLR pathways act as a mechanistic control panel within the TME, balancing acute inflammation and antigen presentation against opposing host mechanisms that restrict viral replication and intratumoral spread (**Figure 2**).

### 4.1 Compartmentalized TLR expression and adaptor architecture (MyD88 vs. TRIF)

TLRs are expressed broadly across the TME by a wide range of immune cells (DCs, macrophages, monocytes, neutrophils, B cells, NK cells) and, in some cancers, by tumor cells themselves. The functional outcome of TLR engagement is dictated by several interlocking variables, including whether receptor localization occurs on the cell surface or within endosomal compartments, as well as the specific responding cell type, the surrounding cytokine milieu, and the baseline immunological state of the tumor (Huang et al., 2022). Surface TLRs (TLR2 and TLR4) sense viral structural proteins, lipoproteins, and damage-associated molecular patterns (DAMPs)-such as HMGB1, heat shock proteins (HSPs), S100 proteins, and extracellular matrix fragments-released during OV-induced immunogenic cell death (ICD) (Chen et al., 2025). Conversely, endosomal TLRs (TLR3, TLR7/8, and TLR9) detect internalized viral nucleic acids, often after the uptake of infected cell debris by APCs. This distribution matters because OV infection generates compartmentalized signals. Tumor cells can initiate local inflammatory cascades through TLR activation, but professional APCs are generally required for effective cross-priming; thus, the most therapeutically relevant TLR activation occurs in DCs and macrophages, which integrate viral sensing with antigen uptake and costimulatory programming (Huang et al., 2022).

### 4.2 The core architecture of Toll-like receptor signaling converges on two fundamental pathways, governed primarily by MyD88-dependent and TRIF-dependent signaling cascades

TLR signaling is classically divided into two distinct adaptor pathways, the first of which is the MyD88-dependent cascade utilized by the majority of receptor types, such as TLR2, TLR4, TLR7, TLR8, and TLR9. Activation of this specific pathway drives the downstream stimulation of NF-κB and MAPKs, which subsequently triggers the robust production of crucial inflammatory cytokines, including TNF, IL-6, and IL-12, heavily associating this axis with inflammatory activation and APC maturation. In contrast, TRIF-dependent signaling, used by TLR3 and partially by TLR4, leads to the activation of IRF3 and the subsequent production of type I interferons (IFN-I) and interferon-stimulated genes (ISGs), which strongly promote antiviral states that restrict viral replication (Troutman et al., 2012). These molecular axes do not operate in isolation; both pathways collaboratively contribute to immune priming, with their net physiological outcome determined by the precise timing, magnitude, and specific cellular context within the TME (Wang et al., 2026).

### 4.3 TLR3 dsRNA sensing and the interferon driven restriction and priming balance

TLR3 functions as a major endosomal sensor of double-stranded RNA (dsRNA), a canonical replication intermediate produced by many RNA viruses and some DNA viruses, and is strongly expressed in DC subsets, macrophages, and certain epithelial or tumor cells (Chen et al., 2021). During oncolytic virotherapy, the therapeutic utility of TLR3 activation lies in its multifaceted capacity to enhance antitumor immunity by driving dendritic cell maturation, upregulating critical costimulatory markers such as CD80 and CD86, and inducing IFN-I production, which substantially augments antigen presentation and cross-priming (Li et al., 2025a). Concurrently, it fuels the synthesis of key chemokines including CXCL10 and CCL5 to actively recruit effector T cells into the tumor microenvironment (Liu et al., 2015). However, excessive TLR3-driven signaling signals primarily through TRIF to activate IRF3 and NF-κB, and the resulting transcriptional program can rapidly induce ISGs in tumor cells and surrounding tissues (Zheng et al., 2021). This restricts viral replication, reducing direct oncolysis and viral transgene expression, which is particularly detrimental for OVs highly sensitive to IFN signaling. The clinical implication of this innate sensing mechanism is tightly linked to the concept of the “interferon window,” a critical temporal framework where an early phase after OV delivery must permit initial viral amplification and tumor cell lysis before transitioning into a later phase where interferon-mediated inflammation actively supports adaptive immune priming (DePeaux and Delgoffe, 2024). Therefore, TLR3 activation may be optimal when spatially localized and temporally constrained, maintaining an initial window for viral spread followed by robust immune priming (Chen et al., 2021).

### 4.4 The ssRNA sensing by TLR7 and 8 and inflammatory programming

TLR7 and TLR8 recognize ssRNA in endosomes and signal through the MyD88 pathway. These receptors are strongly expressed in plasmacytoid DCs (pDCs), which act as major IFN-I producers, and myeloid cells (Bauer et al., 2008). In the OV context, TLR7/8 activation enhances the local inflammatory environment that supports NK and T cell recruitment while strengthening APC licensing (Zhou et al., 2015). This provides a clear mechanistic basis for combining OVs with external TLR7/8 agonists or designing engineered OVs that enhance endosomal RNA sensing. Within this framework, the synthesized IFN-I operates as a critical double-edged sword, given that its ability to robustly enhance antigen presentation is frequently offset by its capacity to rapidly restrict intratumoral viral replication and clear the therapeutic agent prematurely (Snell et al., 2017). Overstimulation risks causing systemic inflammation and accelerated viral clearance. Importantly, in myeloid-dominant tumors, TLR7/8 activation can sometimes produce mixed outcomes if myeloid cells respond to the acute inflammation by generating immunosuppressive mediators as negative feedback (Wu et al., 2025b).

### 4.5 TLR9 CpG DNA sensing and implications for DNA OVs

As a critical intracellular sensor, TLR9 specifically recognizes unmethylated CpG DNA motifs commonly present in microbial genomes. TLR9 is primarily expressed in plasmacytoid dendritic cells and B cells, rendering this pathway particularly relevant for both the deployment of DNA-based oncolytic viruses, such as herpes simplex virus 1 and vaccinia virus, and the strategic implementation of CpG-adjuvant approaches designed to augment antitumor immunity (Krieg, 2007). When the lysis of tumor cells releases viral DNA that is subsequently processed by APCs, TLR9 activation supports IFN-I production, drives DC maturation, upregulates co-stimulatory molecules (CD80/CD86), and improves antigen cross-presentation (Kang et al., 2019). However, its ultimate role may be context-dependent; while some settings suggest that TLR9 signaling enhances anti-tumor immunity, others indicate that strong TLR9-driven antiviral responses can limit viral persistence and blunt direct oncolysis. The net effect likely depends on tumor permissiveness, viral engineering, and whether the primary therapeutic goal is maximal oncolysis or maximal immune priming (Nielsen et al., 2025).

### 4.6 TLR2 and TLR4 sensing viral proteins and DAMPs during OV induced cell death

Surface TLR2 and TLR4 play key roles in detecting viral structural components and released DAMPs generated during tumor destruction. These receptors signal primarily through MyD88 (TLR4 also engages TRIF) to activate NF-κB and inflammatory cytokine production (Qian et al., 2026). During oncolytic virotherapy, this activation substantially drives the functional maturation of macrophages and dendritic cells, concomitantly leading to enhanced phagocytosis, accelerated antigen uptake, and a robust cytokine cascade that supports subsequent T cell priming (DePeaux and Delgoffe, 2024). However, these pathways can also polarize macrophages toward divergent functional fates depending on the cytokine context, meaning they should be interpreted within the broader immunological landscape rather than as universally beneficial (Chi et al., 2025). In a cytokine microenvironment enriched with IL-10, TGF-β, and prostaglandins, TLR2/4 activation can paradoxically reinforce tolerogenic macrophage programs rather than driving pro-inflammatory defenses. This skewed activation state drives the robust production of IL-10, upregulates PD-L1, stimulates arginase 1 induction, and recruits Tregs to reinforce a chronic, immunosuppressive niche that supports tumor progression (Cao et al., 2010). Because macrophages are abundant in many tumors and act as viral scavengers, this compartment represents a formidable barrier to therapeutic success through its dual roles in mediating viral clearance and enforcing local immune suppression; however, it also presents a promising therapeutic opportunity if these plastic cells can be successfully reprogrammed into inflammatory APCs using macrophage modulators (CSF1R blockade, PI3Kγ inhibitors, CD40 agonists) to stabilize a pro-inflammatory state (Jumaniyazova et al., 2025).

### 4.7 TLR signaling and antigen presentation licensing DCs for cross priming

A consistent observation across diverse OV models is that the quality of DC activation determines the magnitude and durability of adaptive immunity, effectively bridging innate sensing and robust adaptive T cell activation. The activation of TLR signaling in dendritic cells drives their functional maturation by upregulating critical costimulatory molecules such as CD80, CD86, and CD40, while concurrently enhancing both MHC class I and class II antigen presentation. This phenotypic shift is accompanied by the robust production of IL-12, which directly supports Th1 polarization and cytotoxic T lymphocyte (CTL) responses, alongside the concomitant induction of CCR7, which orchestrates the directional migration of these matured dendritic cells to draining lymph nodes (Wang et al., 2025). OVs provide both antigen and adjuvant signals to license DCs for cross-priming, but the balance of TLR-driven cytokines is critical. For example, IL-12 supports effector differentiation, whereas excessive IL-10 can suppress priming. Thus, the downstream TLR output signature may be a more informative biomarker than TLR expression alone (Clayton et al., 2025).

### 4.8 The innate sensing paradox in cancer immunotherapy

A defining paradox in OV therapy is that stronger innate antiviral responses present a profound biological trade-off, where robust innate immune recognition is essential to drive effective DC activation, yet this identical intensification inherently triggers potent antiviral mechanisms that restrict viral replication and shorten the oncolytic window (DePeaux and Delgoffe, 2024). This paradox is most evident for IFN-I; it enhances antigen presentation and immune activation, but it also induces an antiviral state that prevents viral spread and recruits myeloid cells that clear the virus and suppress T cells (Odorizzi and Wherry, 2013). This is a frequently observed real-world phenomenon: tumors with intact IFN signaling often restrict OV replication but may still respond if the immune system is successfully primed, whereas IFN-defective tumors permit robust viral replication but fail to generate durable immunity without additional immune stimulation (Huang et al., 2012). Therefore, oncolytic virotherapy efficacy is not a simple equation where more virus or more inflammation automatically equals a better outcome. It depends on the ability to orchestrate a lytic phase that releases antigens and DAMPs, followed by a priming phase that converts this material into durable T cell immunity by optimizing the timing and cellular localization of TLR-driven signals (Davola and Mossman, 2019).

### 4.9 Spatial coordination and crosstalk of DNA sensing: endosomal TLR9 and cytosolic cGAS-STING pathways

For DNA-based oncolytic viruses (OVs)-such as Herpes Simplex Virus (HSV), Adenovirus, and Vaccinia virus-innate immune recognition within the TME serves as a double-edged sword. While it can restrict early viral replication, it is simultaneously the primary driver of the immunogenic “heat” required to reverse local immunosuppression. In modern immuno-oncology, the cyclic GMP-AMP synthase (cGAS)-stimulator of interferon genes (STING) axis is recognized as the principal driver of viral-induced and spontaneous Type I interferon (IFN) production. Rather than acting in isolation, the host cell orchestrates a sophisticated, spatially compartmentalized defense network by coordinating cytosolic cGAS-STING activation with endosomal TLR9 signaling (Xiao et al., 2026).

#### 4.9.1 Compartmentalized DNA recognition and downstream signaling

During viral entry via endocytosis or the phagocytic uptake of viral particles and dying tumor cells by antigen-presenting cells (APCs), viral DNA is exposed within the endolysosomal compartment. Here, TLR9 directly binds to unmethylated CpG DNA motifs characteristic of viral genomes. Upon activation, TLR9 recruits the cytosolic adapter MyD88, forming the myddosome complex. This initiates a signaling cascade that activates tumor necrosis factor receptor-associated factor 6 (TRAF6) and IkappaB kinase alpha (IKKalpha), ultimately driving the nuclear translocation of IRF7 and NF-kappaB to induce rapid transcriptional upregulation of Type I IFNs (alpha/beta) and pro-inflammatory cytokines (Brencicova and Diebold, 2013).

Conversely, when OVs undergo uncoating in the cytoplasm, or when viral replication intermediates and damaged mitochondrial DNA spill into the cytosol, the presence of aberrant cytosolic dsDNA triggers the nucleotidyltransferase cGAS. Upon binding dsDNA in a sequence-independent manner, cGAS undergoes conformational changes to catalyze the synthesis of the secondary messenger cyclic GMP-AMP (2’3’-cGAMP). This cyclic dinucleotide binds to STING, localized at the endoplasmic reticulum (ER) membrane. Activated STING then oligomerizes and translocates to the ER-Golgi intermediate compartment (ERGIC), where it recruits TANK-binding kinase 1 (TBK1). TBK1 phosphorylates both STING and the transcription factor IRF3. Phosphorylated IRF3 dimerizes and translocates to the nucleus, where it synergizes with NF-kappaB to robustly drive the expression of Type I IFNs and interferon-stimulated genes (ISGs) (Guo et al., 2020; Liu and Xu, 2025).

#### 4.9.2 Mechanistic crosstalk and amplification loops

The interface between the endosomal TLR9 and cytosolic cGAS-STING pathways represents a highly coordinated crosstalk that amplifies the innate immune landscape of the TME. Rather than operating as redundant redundant systems, these pathways function sequentially and synergistically. First, the primary wave of Type I IFNs generated by early TLR9 activation in dendritic cells and macrophages can upregulate the baseline expression of cGAS, STING, and TBK1, which are themselves interferon-stimulated genes. This primes the cell, lowering the threshold required for cytosolic DNA sensing as the viral infection progresses. Second, recent evidence indicates a critical crossover in secondary messenger signaling. The 2’3’-cGAMP generated by tumor cells undergoing active replication of DNA OVs can be packaged into budding virions or transferred directly into adjacent non-infected cells and APCs via gap junctions or specialized transporters (such as SLC19A1). Once internalized by neighboring immune cells, this extracellular cGAMP can directly engage STING independently of cytosolic DNA uncoating, overlapping with the active TLR9 signaling triggered by the endocytosis of intact viral particles (Deb et al., 2020).

Furthermore, the downstream signaling nodes of both pathways converge at the level of the IKK complex and TBK1, enabling a cooperative transcriptional network where IRF3, IRF7, and NF-kappaB bind simultaneously to the IFN-beta enhanceosome. This dual-axis activation ensures a robust, sustained production of Type I IFNs, which is critical for the recruitment and activation of tumor-infiltrating NK cells and cytotoxic CD8^+^ T cells (Yum et al., 2021).

#### 4.9.3 Therapeutic implications for DNA oncolytic virotherapy

Understanding this spatial coordination is paramount for the design of modern DNA OVs. Many successful clinical-stage OVs are strategically engineered to modulate this crosstalk; for instance, deleting viral virulence factors that naturally antagonize the STING or TBK1 pathway (such as HSV ICP0 or ICP34.5) allows uninhibited cGAS-STING activation, converting a “cold” stroma into a highly inflamed microenvironment. Consequently, maximizing the synergy between endosomal TLR9 and cytosolic cGAS-STING sensing represents a cornerstone strategy for optimizing the immunogenicity of DNA oncolytic virotherapy (Sibal et al., 2024).

#### 4.9.4 Interplay between TLRs and other PRRs including RIG-I, MDA5, cGAS-STING, and inflammasomes

Although this synthesis emphasizes TLRs, OV sensing involves multiple pattern recognition receptors operating as part of a larger sensing network. Cytosolic sensors such as RIG-I and MDA5 detect viral RNA and contribute to IRF3/7 activation, while DNA sensing through cGAS-STING can be vital for DNA viruses and for host DNA released during cell death. Concurrently, inflammasome activation can contribute to IL-1β and IL-18 production, shaping both innate and adaptive immunity (Liu et al., 2025b). In some settings, TLR activation may be redundant with cytosolic sensing; in others, TLR signaling remains essential for APC licensing. This complexity reinforces the need for mechanistic biomarkers and careful interpretation of immune signatures in OV-treated patients (Duan et al., 2022).

### 4.10 Crosstalk between TLR signaling and immune tolerance pathways

Sustained TLR hyperactivation frequently triggers compensatory immune checkpoint pathways within the TME that reinforce tolerance (Hoden et al., 2022). First, TLR activation can induce autocrine and paracrine IL-10 and TGF-β feedback in macrophages and DCs, which naturally limits tissue damage but creates a setting where TLR signals are captured into tolerogenic programs within pre-existing suppressive tumors (Norollahi et al., 2025). Second, type I IFN and NF-κB signaling can upregulate PD-L1 on tumor cells and myeloid cells, explaining why OV monotherapy sometimes fails after an initial inflammatory burst, while simultaneously establishing a mechanistic basis for OV synergy with PD-1/PD-L1 blockade (Antonangeli et al., 2020). Finally, TLR-driven chemokines risk recruiting inflammatory monocytes that subsequently differentiate into suppressive TAMs; without proper reprogramming, the tumor microenvironment may transition from an acute inflammatory state to a chronic, suppressive state (Qin et al., 2023).

### 4.11 Strategies to tune TLR pathways in OV therapy

Multiple translational approaches have been explored to exploit TLR signaling while avoiding premature viral clearance. These include engineering OVs to express specific immunostimulatory cytokines (such as GM-CSF and IL-12), deleting viral genes that strongly trigger innate sensing to establish IFN-resistant viral replication, or encoding immune checkpoint modulators locally (Omolekan et al., 2025). Optimizing clinical outcomes relies heavily on strategic companion scheduling and dosing, such as utilizing repeated intratumoral injections, implementing prime-boost schedules, or delaying external TLR agonist administration to allow an initial window for maximum viral amplification before inducing the systemic vaccine effect (Mondal et al., 2025). Intratumoral delivery reduces systemic IFN activation and allows a higher local viral load to accumulate, increasing antigen release and DC recruitment (Omolekan et al., 2025). Furthermore, co-administered chemotherapy can create a transient immunological reset that changes how TLR signals are interpreted by depleting suppressive myeloid cells, increasing antigen availability, inducing ICD, and altering DC activation thresholds. This sets up the mechanistic basis for OV-chemotherapy synergy, which is the focus of the next major section (Ding et al., 2014).

## 5 Chemotherapy-induced remodeling of oncolytic virotherapy responses

Chemotherapy remains a standard of care backbone for many cancers and is frequently used in the same clinical contexts where OVs are being tested. While historically regarded as immunosuppressive, modern immuno-oncology has clarified that chemotherapy can exert strong immunomodulatory effects. Importantly, these effects are not uniform; they depend on the specific agent, dose intensity, timing, and tumor context (Sordo-Bahamonde et al., 2023). Within the context of oncolytic virotherapy, the concurrent administration of chemotherapy exerts a multi-faceted influence that simultaneously shapes viral fitness and orchestrates immune priming. Regarding viral dynamics, cytotoxic agents can directly alter viral replication, intratumoral spread, and overall persistence within the malignant tissue, depending on the specific drug mechanism and timing. Concurrently, this chemotherapy-induced modulation extends to host immunity by shifting dendritic cell function, altering T cell availability, and reshaping suppressor cell dynamics, which collectively dictate whether the microenvironment shifts toward productive antitumor immunity or sustained tolerance. Thus, OV-chemotherapy combinations should be understood as immunological co-therapies rather than merely cytotoxic add-ons (Fayyad-Kazan et al., 2026).

### 5.1 Chemotherapy-induced lymphopenia exerts a paradoxical effect on oncolytic virotherapy by removing immune barriers to enhance viral replication while simultaneously constraining the development of robust anti-tumor immunity

Many cytotoxic regimens cause lymphopenia, including depletion of CD8^+^ T cells, NK cells, and B cells. This is typically detrimental for durable anti-tumor immunity, particularly for OV platforms whose long-term efficacy depends on T cell priming. However, chemotherapy-induced lymphopenia can also substantially reduce antiviral immune clearance, a phenomenon that potentially permits higher intratumoral viral loads, prolonged viral replication, and increased oncolysis alongside a more extensive release of tumor-associated antigens. This biological shift creates a profound therapeutic trade-off where the co-administered chemotherapy may markedly improve early oncolysis and direct viral destruction of the tumor, but simultaneously impairs the host immune system’s long-term ability to successfully convert these liberated tumor antigens into durable immunological memory (Imani et al., 2025). Therefore, the timing of chemotherapy relative to OV delivery is critical. In many cases, chemotherapy may be more beneficial when given before oncolytic virotherapy to reduce suppressive cells and tumor burden, or after viral delivery to avoid blunting early immune priming.

### 5.2 Immunogenic cell death serves as a mechanistic bridge that connects chemotherapy with oncolytic virotherapy induced vaccination to drive powerful anti-tumor immunity

A major mechanistic rationale for combining chemotherapy with oncolytic viruses is the amplification of ICD. Several chemotherapeutics induce hallmarks of this process, including calreticulin exposure, ATP release, HMGB1 release, and type I interferon induction, while oncolytic viruses simultaneously drive complementary inflammatory death through viral replication and lysis. OVs also induce ICD-like inflammatory death through viral replication and lysis. When combined, these therapies can converge to enhance DAMP release, increase antigen availability, and drive stronger dendritic cell activation and cross presentation. This convergence is especially relevant for tumors that are immune-desert or poorly inflamed at baseline. In such settings, the combination can shift the tumor toward an inflamed phenotype capable of supporting T cell infiltration (Cao et al., 2025).

### 5.3 Chemotherapy effects on antigen presentation and DC competence

Chemotherapy can modulate antigen presentation at multiple levels by increasing tumor antigen release through apoptosis or necrosis, upregulating MHC-I in some contexts, and reducing suppressive cytokines through the depletion of tumor cells and stromal sources. However, some regimens can impair DC function, particularly when administered at high dose intensity. Since DC cross-presentation is central to OV-mediated *in situ* vaccination, DC competence becomes a key variable determining whether combination therapy yields synergy or antagonism (Li et al., 2021a).

### 5.4 The myeloid compartment in OV-chemotherapy synergy

#### 5.4.1 Myeloid-derived suppressor cells

Many tumors characteristically expand the population of myeloid-derived suppressor cells, which aggressively suppress T cell function through diverse biochemical mechanisms, including the production of arginase 1, the upregulation of inducible nitric oxide synthase, the generation of reactive oxygen species, and the localized depletion of essential amino acids required for T cell proliferation. Certain chemotherapies can reduce MDSCs, creating a more permissive environment for OV-induced T cell priming (Dash et al., 2026).

#### 5.4.2 Tumor-associated macrophages

TAMs can clear viral particles, restrict viral spread, secrete IL-10 and TGF-β, and promote angiogenesis and tumor repair. Some chemotherapeutic agents can transiently deplete these macrophages or shift their polarization toward inflammatory states, which may ultimately enhance oncolytic virotherapy efficacy. Conversely, rebound myelopoiesis after chemotherapy can restore suppressive macrophage populations, creating a window of vulnerability where OV therapy must be timed carefully (Yang et al., 2020).

### 5.5 Chemotherapy interacts directly with TLR signaling pathways to reset innate immune thresholds within the tumor microenvironment

Chemotherapy exerts a profound impact on the TME by altering TLR responses in both immune and tumor cells through distinct molecular mechanisms. Mechanistically, cytotoxic treatments increase nucleic acid release and enhance endosomal delivery, thereby significantly amplifying the stimulation of TLR 3, 7, and 9. Concurrently, chemotherapy promotes the robust release of damage-associated molecular patterns to enhance TLR 2 and 4 activation, while fundamentally altering baseline cytokine profiles to shift overall receptor outputs toward either inflammatory or tolerogenic states depending on the specific therapeutic context. Therefore, chemotherapy can reprogram the TLR paradox. Under optimal conditions, it may allow for sufficient viral replication followed by robust dendritic cell activation and priming. Conversely, under suboptimal conditions, it may instead amplify IL-10 feedback, PD-L1 induction, and myeloid-driven immune tolerance (Yi et al., 2023).

### 5.6 Strategic scheduling principles and dosing sequences

A consistent insight across OV combination studies is that timing often matters more than the specific chemotherapy agent (Cheng et al., 2025).

### 5.7 Mechanisms of antagonism and therapeutic interference

Not all combinations are synergistic. Antagonism can occur via the depletion of dendritic cells and effector lymphocytes, impairment of antigen presentation, increased vascular damage limiting viral delivery, and excessive inflammation causing premature viral clearance. Clinically, warning signs of antagonism may include minimal intratumoral viral replication, low induction of CXCL10 and CCL5, a lack of dendritic cell maturation markers, and a rapid rebound of suppressive myeloid cells (Gregori et al., 2025).

## 6 Mechanistic basis of OV-chemotherapy synergy

### 6.1 Sequential orchestration involves a coordinated cascade where initial tumor lysis and widespread antigen release directly pave the way for robust, systemic immune priming

**Figure 3:**
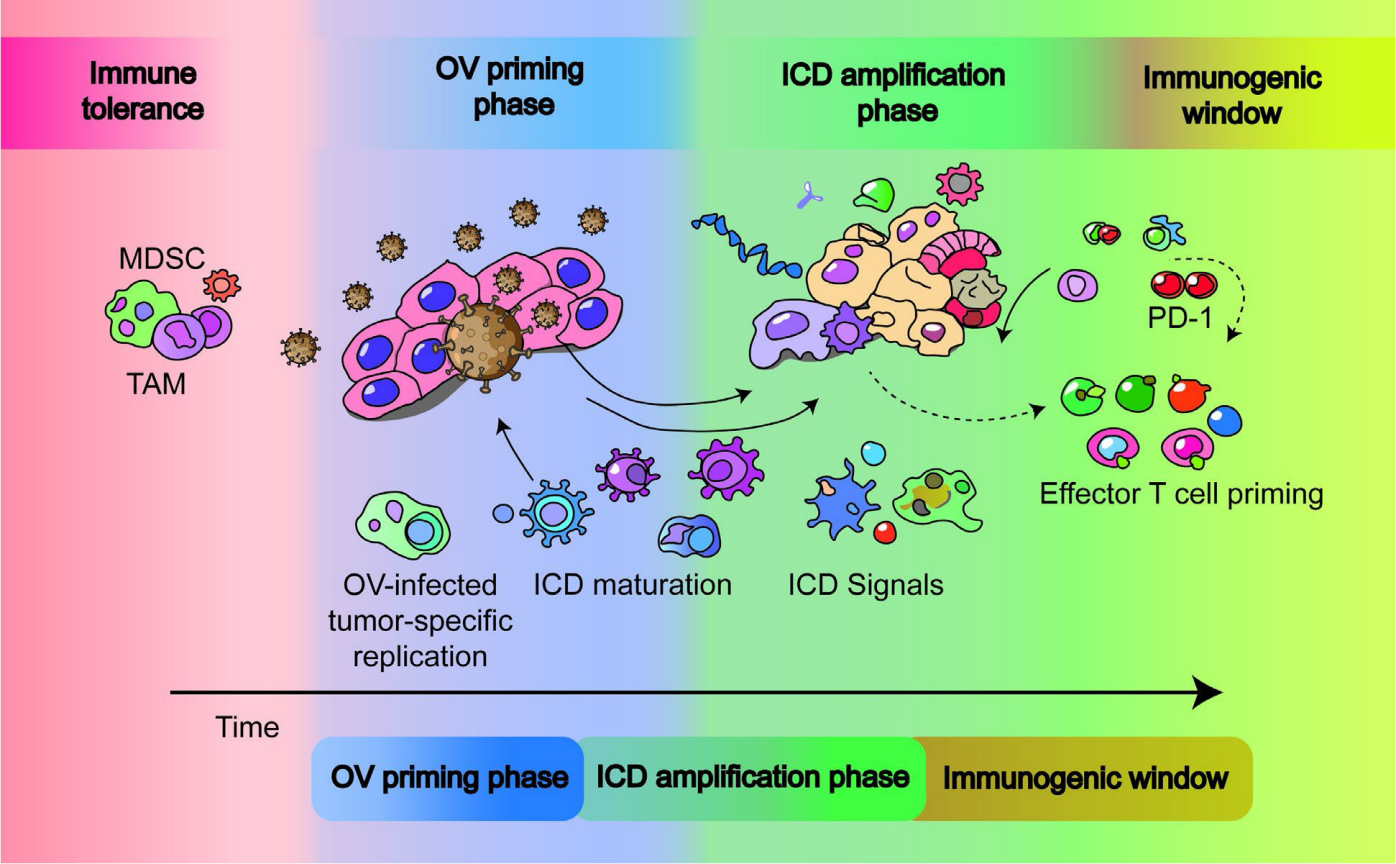
OV-chemotherapy synergy timeline. Immune tolerance (baseline suppressive TME), OV priming and ICD amplification (OV infection, DAMP release, DC activation), Immunogenic window (effector T cell priming, PD-1/PD-L1 upregulation, optimal anti-tumor immunity).

OVs and chemotherapy converge on overlapping mechanisms of immunogenic cell death. Chemotherapy can enhance viral-mediated immunogenic cell death by increasing tumor cell susceptibility to infection, promoting calreticulin exposure, driving ATP and HMGB1 release, and enhancing dendritic cell uptake and cross-presentation of tumor antigens. For instance, anthracyclines and oxaliplatin have been shown to amplify virus-induced immunogenic cell death, leading to more robust effector T cell recruitment in preclinical tumor models. Temporal sequencing remains critical, as viral-mediated antigen release followed by chemotherapy-induced DAMP amplification produces a larger immunogenic window that significantly improves T cell priming (**Figure 3**).

### 6.2 Modulation of the tumor microenvironment

Chemotherapy actively modulates the TME by inducing a transient depletion of immunosuppressive compartments, such as myeloid-derived suppressor cells (MDSCs), tumor-associated macrophages (TAMs), and regulatory T cells (Tregs). Building upon this altered immunological landscape, subsequent OV infection further reprograms macrophages and dendritic cells through TLR-dependent pathways. This sequential remodeling not only alleviates localized immune suppression but also actively drives the maturation of antigen-presenting cells within the niche. The combination often converts an immune-excluded or cold TME into an inflamed hot TME conducive to cytotoxic T cell infiltration. Key cytokine shifts include reduced IL-10/TGF-β and increased type I IFNs, IL-12, and CXCL9/10 (Li et al., 2021b).

### 6.3 Checkpoint modulation and synergy with immunotherapy

OVs and chemotherapy combinations frequently induce adaptive immune resistance, such as PD-L1 upregulation on tumor and myeloid cells. This adaptive response provides a strong rationale for triple therapy, where the oncolytic virus induces tumor lysis and TLR-driven immune priming, chemotherapy amplifies immunogenic cell death and reduces suppressive cells, and checkpoint blockade prevents premature T cell exhaustion, thereby maximizing overall antitumor efficacy. Clinical correlative studies suggest that OV-chemotherapy therapy creates a T cell inflamed phenotype, which is predictive of response to PD-1/PD-L1 inhibitors (Gregori et al., 2025).

### 6.4 Tuning of innate immunity

Chemotherapy influences TLR-mediated innate immune sensing, as enhanced nucleic acid availability directly stimulates TLR3, TLR7, and TLR9 in dendritic cells, thereby improving type I interferon and downstream cytokine responses. Modulation of DAMP-sensing TLR2/4 shifts TAM polarization toward inflammatory phenotypes. Timing is crucial to preserve early viral replication before robust IFN responses constrain OV amplification. Preclinical models show that carefully timed chemotherapy permits sufficient OV spread while preserving innate immune activation necessary for adaptive priming (Goradel et al., 2021).

## 7 Clinical evidence across OV platforms

### 7.1 Herpes simplex virus-based OVs

In clinical settings, the combination of T-VEC and chemotherapy in melanoma patients significantly enhances intratumoral T-cell infiltration alongside antigen-specific CD8^+^ T-cell responses. This observed clinical efficacy correlates closely with insights from preclinical studies, which indicate that taxanes and platinum compounds synergize with herpes simplex virus (HSV)-based OVs through the amplification of ICD (Larocca et al., 2020).

### 7.2 Adenovirus-based OVs

Adenovirus platforms have been combined with gemcitabine in pancreatic cancer models. Gemcitabine reduces MDSCs and Tregs, increasing OV spread and CD8^+^ T cell priming. Early-phase clinical trials show partial responses and immune activation consistent with preclinical predictions (Eriksson et al., 2016).

### 7.3 Reovirus and vaccinia virus OVs

Reovirus exhibits synergistic effects with cyclophosphamide or cisplatin in sarcoma and ovarian cancer models through suppression of regulatory myeloid compartments, amplification of TLR3/IFN-driven dendritic cell activation, and enhancement of effector T cell priming. Vaccinia-based OVs combined with 5-fluorouracil or oxaliplatin demonstrate similar synergy, including tumor regression and prolonged survival in murine models (Fayyad-Kazan et al., 2026).

### 7.4 Key translational lessons

Agent specificity matters because DNA and RNA OVs differ significantly in their TLR engagement and chemotherapy compatibility. Sequence and timing remain critical, as optimal synergy often requires viral priming before or concurrent with chemotherapy administration. Furthermore, immune monitoring is highly predictive; effector CD8^+^ T cell expansion, interferon-stimulated gene induction, and tumor-associated macrophage reprogramming correlate strongly with therapeutic response (Fan et al., 2026b).

## 8 Clinical translation, trial failures, and translational barriers

Despite the compelling preclinical efficacy and elegant mechanistic orchestrations demonstrated by various OVs, their clinical translation has been fraught with substantial bottlenecks. To date, talimogene laherparepvec (T-VEC) remains the only FDA-approved OV for advanced melanoma, serving as a proof-of-concept but also highlighting the steep gradient between preclinical promise and real-world clinical outcomes. The underwhelming performance of numerous OV candidates in late-stage clinical trials underscores a profound disconnect between simplified laboratory models and the complex realities of human oncology (Li et al., 2022).

### 8.1 Lessons from advanced trial underperformance and failures

A critical evaluation of recent Phase III trial failures reveals that robust intratumoral viral replication in mice rarely translates to a durable clinical response in humans. A primary example is Pexa-Vec (JX-594), an oncolytic vaccinia virus. In the Phase III PHOCUS trial for advanced hepatocellular carcinoma (HCC), Pexa-Vec combined with sorafenib failed to improve overall survival compared to sorafenib alone, leading to early termination following an interim futility analysis (Abou-Alfa et al., 2024).

Preclinical evaluation predominantly relies on immunodeficient or syngeneic murine models. These models possess accelerated tumor kinetics and lack the intricate, long-term co-evolution of human tumors and the immune system. Furthermore, murine cells often exhibit different IFN pathway sensitivities compared to human cells, leading to overestimations of viral replication and oncolysis *in vitro*. While intratumoral injection (like T-VEC) circumvents systemic clearance, it is impractical for deeply seated metastatic lesions. Systemic (intravenous) administration remains the holy grail for widespread metastasis, yet it faces immediate neutralization by pre-existing or treatment-induced neutralizing antibodies (NAbs), complement activation, and splenic/hepatic sequestration. Consequently, an infinitesimal fraction of the initial viral inoculum successfully reaches the tumor parenchyma (Zhou et al., 2026).

### 8.2 Patient heterogeneity and host-virus kinetics

Patient heterogeneity represents a paramount barrier to uniform OV efficacy. Human populations possess highly variable baseline antiviral immunity due to prior natural exposures or vaccinations (e.g., prior exposure to Adenovirus, Herpes Simplex Virus, or Coxsackievirus) (Zaiss et al., 2009).

In patients with high baseline anti-vector immunity, systemically delivered OVs are cleared almost instantaneously. Conversely, even in naive patients, the initial viral doses inevitably trigger a robust secondary immune response. This creates a narrow therapeutic window where the host immune system prematurely eradicates the virus before significant tumor oncolysis can occur. Translation is further complicated by the dichotomy of the immune response. For an OV to succeed, it must induce *antitumor* immunity (by releasing tumor-associated antigens during immunogenic cell death). However, the host simultaneously mounts a potent *antiviral* immune response. Balancing these two arms-suppressing the antiviral response long enough for replication while boosting the antitumor response-remains an unresolved clinical challenge (Shin et al., 2021).

### 8.3 Physical barriers and the immunosuppressive microenvironment

Human Desmoplastic tumors (such as pancreatic ductal adenocarcinoma) exhibit dense collagen networks and high interstitial fluid pressure (IFP). This physical barrier impedes the intratumoral lateral spread of the virus, confining viral infection to localized pockets near the injection site and preventing complete tumor debulking. Advanced human tumors are heavily infiltrated with myeloid-derived suppressor cells (MDSCs), Tregs, and M2-polarized macrophages. This hostile microenvironment rapidly dampens the alarmone signals and pro-inflammatory cytokines triggered by viral infection, effectively quenching the “heat” generated by the virus and preventing the recruitment of functional CD8^+^ tumor-infiltrating immune cells (TIICs) (Provenzano et al., 2012).

### 8.4 Overcoming barriers via next-generation rational combinations

Recognizing these barriers, the paradigm has shifted from OV monotherapy to rational combination regimens, particularly with immune checkpoint inhibitors (ICIs). For instance, the combination of CG0070 (an oncolytic adenovirus) with pembrolizumab in the Phase II CORE-001 trial for non-muscle invasive bladder cancer (NMIBC) demonstrated a remarkable 85% complete response rate at 12 months. Similarly, DNX-2401 (tasadenoturev) combined with pembrolizumab in recurrent glioblastoma showed durable survival benefits in a subset of patients. By utilizing the OV as an *in situ* vaccine to “turn cold tumors hot,” followed by ICIs to sustain the T-cell response, current clinical strategies are beginning to dismantle the barriers that caused early-generation trial failures (Adashek et al., 2025).

## 9 Mechanistic heterogeneity of DNA and RNA oncolytic platforms

The therapeutic efficacy of oncolytic immunotherapy is fundamentally governed by the structural and genomic diversity of the underlying viral platform. Rather than acting as a monolithic class, DNA- and RNA-based OVs engage distinct cellular entry receptors, activate divergent PRRs, and utilize idiosyncratic native mechanisms to evade host clearance, which collectively dictate the kinetics of tumor oncolysis and immune remodeling (Abdalhamed et al., 2022).

### 9.1 Innate immune engagement and nucleic acid sensing pathways

The primary divergence between DNA and RNA OVs lies in the spatial and biochemical orchestration of host PRR activation. Large dsDNA viruses, such as HSV-1, Adenovirus (Ad), and Vaccinia Virus (VV), typically exhibit a protracted replication cycle involving nuclear or specialized cytoplasmic hubs. During cell entry and uncoating, these platforms primarily engage endosomal TLR9 and the cytosolic cyclic GMP-AMP synthase (cGAS)-STING pathway. Activation of the cGAS-STING axis triggers the phosphorylation of IRF3 via TBK1, initiating a steady, sustained induction of Type I IFNs and downstream interferon-stimulated genes (ISGs). This creates a prolonged therapeutic window, allowing the virus to replicate substantially within the tumor core before host clearance occurs (Cai et al., 2021).

Conversely, single-stranded or double-stranded RNA viruses, including Vesicular Stomatitis Virus (VSV), Measles Virus (MV), and Reovirus, undergo rapid, high-copy replication exclusively within the host cell cytoplasm. The generation of viral replication intermediates-specifically dsRNA or 5′-triphosphate RNA-acts as an immediate, aggressive danger signal. These structures are rapidly sensed by endosomal TLR3 and TLR7/8, alongside the cytosolic RIG-I-like receptor (RLR) family, including Retinoic Acid-Inducible Gene I (RIG-I) and Melanoma Differentiation-Associated Protein 5 (MDA5). This profound RLR engagement bypasses nuclear transport dependencies, culminating in an acute, explosive “interferon storm.” While this intense pro-inflammatory burst is highly efficient at rapidly “turning cold tumors hot” by converting the local immunosuppressive microenvironment, it simultaneously accelerates antiviral host immunity, occasionally truncating the window available for direct viral oncolysis (Inoue, 2026).

### 9.2 Virus-specific tropism and native immune evasion strategies

HSV-1 and Adenovirus: HSV-1 utilizes its envelope glycoproteins to selectively bind to cell-surface Nectin-1 and Herpesvirus Entry Mediator (HVEM), molecules frequently upregulated in malignancies of neuroectodermal origin. To survive host immune surveillance, wild-type HSV-1 deploys its ICP47 protein, which binds competitively to the Transporter associated with Antigen Processing (TAP), preventing peptide loading onto Major Histocompatibility Complex Class I (MHC-I) and shielding infected cells from early CD8^+^ T-cell destruction. Similarly, Adenovirus targets the Coxsackie-Adenovirus Receptor (CAR) or CD46 for internalization via its fiber and knob structures. It relies heavily on its E3 transcription unit to downregulate cell-surface MHC-I and actively subvert tumor necrosis factor-alpha (TNF-alpha)-mediated apoptosis (Scanlan et al., 2022).

Among RNA vectors, VSV exhibits an exceptionally broad cellular tropism by utilizing its single envelope glycoprotein (VSV-G) to engage the nearly ubiquitous Low-Density Lipoprotein Receptor (LDLR). However, VSV is highly sensitive to Type I IFNs, making its tumor-selectivity dependent on defects in the IFN pathway common to cancer cells. To counteract this, wild-type VSV utilizes its Matrix (M) protein to globally block host nuclear-cytoplasmic mRNA transport, silencing host IFN synthesis. Measles virus exploits CD46 (an overexpressed complement regulatory protein on many human tumors) or SLAM for cell fusion and entry, utilizing its native V and C proteins to inhibit STAT1/2 phosphorylation, thereby temporarily disabling downstream JAK-STAT interferon signaling within the infected tumor microenvironment (Hastie et al., 2013).

## 10 Engineering paradigms: unarmed vs. armed OVs

The evolution of oncolytic virotherapy is marked by a structural shift from first-generation “unarmed” platforms to next-generation “armed” genetic delivery systems, a transition driven by the need to overcome the physical and immunological barriers of the tumor microenvironment (TME) (Wong et al., 2023).

### 10.1 Unarmed OVs: direct oncolysis and intrinsic selectivity

First-generation, or “unarmed,” OVs rely strictly on natural or engineered mutations to selectively replicate in and lyse malignant cells without carrying foreign therapeutic transgenes. For instance, early-generation HSV vectors were rendered tumor-selective through the deletion of the ICP34.5 gene (neurovirulence factor) and the ICP47 gene. Deletion of ICP34.5 restricts viral replication exclusively to tumor cells with constitutively active Ras/MEK/ERK signaling or compromised double-stranded RNA-dependent protein kinase (PKR) pathways, leaving healthy post-mitotic neurons unharmed. Unarmed OVs exert their therapeutic benefit predominantly through direct cytopathic oncolysis-replicating until the host cell bursts-which releases native tumor-associated antigens (TAAs) and damage-associated molecular patterns (DAMPs) into the stroma. While safe and conceptually elegant, unarmed OVs frequently face clinical limitations because direct viral lysis alone is often insufficient to dismantle the heavily entrenched, dominant immunosuppressive architecture of advanced human metastases (Sasso et al., 2020).

### 10.2 Armed OVs: transgene-mediated microenvironmental remodeling

To transcend the limits of simple lysis, modern synthetic virology leverages OVs as highly programmable, replication-competent gene delivery vehicles. The large packaging capacity of DNA viruses (ranging from ~8 kb in Adenoviruses to over 30-40 kb in HSV and Vaccinia) makes them uniquely suited for this “arming” paradigm, whereas RNA viruses possess tighter cargo constraints (~4.5-5 kb for VSV and Measles) but replicate with high velocity (Sasso et al., 2020).

The most clinically validated approach involves inserting cytokine genes to amplify antitumor immunity. T-VEC represents the archetype, armed with human Granulocyte-Macrophage Colony-Stimulating Factor (GM-CSF). Upon cell lysis, locally secreted GM-CSF recruits and differentiates regional dendritic cells and antigen-presenting cells (APCs), optimizing the priming of tumor-specific CD8^+^ T cells. Current clinical strategies have advanced to arming OVs with potent interleukins (e.g., IL-12, IL-15) or membrane-bound costimulatory ligands (e.g., OX40L, 4-1BBL), directly sustaining T-cell activation within the TME (Ferrucci et al., 2021).

To combat physical delivery barriers, OVs have been rationally armed with matrix-degrading enzymes, such as collagenase, hyaluronidase, or decorin. Upon local expression, these enzymes selectively degrade the dense desmoplastic collagen network characteristic of solid tumors (e.g., pancreatic and breast cancers). This degradation reduces intratumoral interstitial fluid pressure (IFP), significantly enhancing both the lateral spread of the virus throughout the tumor mass and the physical infiltration of newly recruited tumor-infiltrating immune cells (TIICs) (Gong et al., 2025).

OVs can also be armed with metabolic “suicide genes,” such as Herpes Simplex Virus Thymidine Kinase (HSV-TK) or Cytosine Deaminase (CD). When expressed locally within the infected tumor bed, these enzymes convert systematically administered, non-toxic prodrugs (e.g., ganciclovir or 5-fluorocytosine) into highly cytotoxic metabolites (ganciclovir triphosphate or 5-fluorouracil). This dual approach achieves a potent “bystander effect,” eradicating neighboring uninfected tumor cells and tumor-associated endothelial structures, effectively combining targeted viral oncolysis with localized chemotherapy (Qi et al., 2022).

## 11 Translational framework: tuning TLRs in oncolytic virotherapy

The therapeutic efficacy of oncolytic virotherapy relies on a delicate equilibrium between viral replication, localized tumor lysis, and the subsequent induction of systemic anti-tumor immunity. Toll-like receptors (TLRs) serve as critical molecular fulcrums in this process. However, leveraging TLR signaling in OV regimens requires moving beyond isolated receptor pathways toward a holistic, translational framework that accounts for tumor heterogeneity, predictive biomarkers, and precise pharmacological modulation (Garmaroudi et al., 2022).

### 11.1 Divergent net effects across distinct tumor microenvironments

The net immunological outcome of TLR activation is not uniform; rather, it is strictly context-dependent and dictated by the baseline tumor microenvironment (TME). In immunologically “hot” tumors (e.g., melanoma, non-small cell lung cancer) characterized by high baseline leukocyte infiltration, TLR activation (particularly TLR3 and TLR7/8) acts synergistically with OV-mediated lysis. It accelerates DC maturation, enhances cross-presentation of tumor-associated antigens (TAAs), and shifts the TME toward a robust Th1-polarized, anti-tumor state (Quail and Joyce, 2026).

Conversely, in “cold” or highly immunosuppressive TMEs (e.g., pancreatic ductal adenocarcinoma, glioblastoma), unguided TLR activation can lead to divergent, detrimental net effects. Chronic activation of TLR2/4 or TLR9 by viral components and DAMPs can inadvertently recruit MDSCs and promote a pro-tumorigenic inflammatory cascade that facilitates immune evasion and tumor cell survival. Therefore, the net effect of TLR engagement post-OV therapy fluctuates between potentiation and antagonism, determined primarily by the specific tumor type and its anatomical immune landscape (Spinetti et al., 2016).

### 11.2 TLR expression signatures as predictive biomarkers

To mitigate these divergent outcomes, baseline TLR expression profiles offer significant potential as predictive biomarkers for patient stratification. Rather than evaluating single receptor levels, mapping comprehensive “TLR expression signatures” via transcriptomic profiling can predict how a specific patient’s TME will respond to an OV cocktail (Ying et al., 2023).

For instance, tumors exhibiting a baseline signature enriched in endosomal TLRs (TLR3, TLR7, and TLR9) alongside high Type I IFN response genes serve as prime candidates for OV monotherapy or OV combined with targeted TLR agonists, as they possess the intrinsic machinery to maximize viral-induced danger signaling. Conversely, a baseline signature dominated by surface TLRs (TLR2/4) coupled with elevated chronic inflammatory markers (e.g., IL-6, TNF-alpha) signals a high risk of driving immune exhaustion. Utilizing these expression signatures prior to therapy allows for precision patient selection, ensuring that combinations are deployed only in cohorts where the net signaling cascade tilts toward robust immune activation (Thomas et al., 2021).

### 11.3 Pharmacological tuning: rational agonist/antagonist scheduling in OV combos

Harnessing the full potential of TLRs in virotherapy ultimately requires active, time-resolved pharmacological tuning using specific small-molecule modulators. The key to successful coordination lies in the chronological scheduling of these agents relative to OV administration (Wang et al., 2020).

To turn a “cold” tumor “hot,” synthetic TLR3 agonists (such as Poly(I:C) or Rintatolimod) or TLR9 agonists (CpG oligonucleotides) can be administered either alongside or shortly after OV delivery. This co-administration mimics a profound, multi-pronged pathogen assault, rapidly accelerating the activation of NK cells and cytotoxic T lymphocytes (CTLs) toward newly liberated TAAs, thereby maximizing the “abscopal effect” in distant metastases (Rolfo et al., 2023).

Conversely, if hyper-activated TLR signaling triggers premature viral clearance by the host’s innate antiviral responses (e.g., excessive early Type I IFN production restricting OV replication), the transient application of TLR antagonists (e.g., specific TLR4 or TLR7/8 inhibitors) during the initial viral replication window can temporarily shield the virus. This pharmacological deceleration permits robust intra-tumoral viral amplification and oncolysis before the subsequent wave of adaptive immunity is intentionally unleashed (Lester and Li, 2014).

Through such precise pharmacological tuning-balancing dynamic agonism to drive adaptive immunity with targeted antagonism to protect viral kinetics-the synergy between OVs and TLR modulators can be personalized to overcome the unique evolutionary defenses of diverse human malignancies (Cao et al., 2025).

## 12 Emerging frontiers in OV-based combinatorial therapies

To fully realize the potential of OV-based chemoimmunotherapy, recent research has pivoted toward integrating OVs with other cutting-edge therapeutic modalities and leveraging advanced analytical platforms. These emerging strategies aim to bypass the physical and immunological barriers of the TME and drive durable clinical responses (Volovat et al., 2024).

### 12.1 Harnessing OV-microbiome interactions and vaccine combinations

A rapidly growing body of evidence highlights a complex, bidirectional relationship between OVs and the host microbiome. The gut and intratumoral microbiota heavily influence systemic immune baseline tones; consequently, combining OVs with microbiota-modulating agents (such as specific probiotics or fecal microbiota transplantation) can prime host dendritic cells and enhance the downstream trafficking of tumor-reactive T cells following viral oncolysis. Simultaneously, OVs are being strategically paired with personalized neoantigen vaccines to address tumor heterogeneity. While neoantigen vaccines precisely expand the host’s T-cell receptor (TCR) repertoire against patient-specific clonal mutations, the co-administration of OVs provides the essential adjuvant effect-inducing local inflammation, liberating cryptic antigens through viral lysis, and converting immunologically “cold” tumors into “hot” targets highly receptive to vaccine-induced T cells (Liu et al., 2024).

### 12.2 Synergies with bispecific antibodies and CAR-T cell therapies

Next-generation viral engineering has also enabled OVs to serve as localized delivery vehicles for complex biologics, effectively mitigating systemic toxicities. A premier example is the development of OVs armed with bispecific antibodies, such as bi-specific T-cell engagers (BiTEs). When these engineered OVs infect tumor cells, they drive the local expression of BiTEs that simultaneously bind tumor-associated antigens and CD3 on T cells, mechanically bypassing the requirement for major histocompatibility complex (MHC) class I presentation. Furthermore, OVs exhibit powerful synergies with chimeric antigen receptor (CAR)-T cell therapies. Solid tumors typically resist CAR-T trafficking due to dense extracellular matrices and immunosuppressive signaling. OVs engineered to secrete chemokines (e.g., CXCL9/10) or matrix-degrading enzymes effectively pave the way for CAR-T cell infiltration, while viral-mediated immunogenic cell death (ICD) relieves local immunosuppression, significantly boosting CAR-T cell persistence and operational longevity within the TME (Rahman and McFadden, 2021).

### 12.3 Mapping TME remodeling via single-cell and multi-omics technologies

Characterizing the intricate, dynamic remodeling of the TME following OV-based chemoimmunotherapy requires resolutions beyond traditional bulk sequencing. The integration of single-cell RNA sequencing (scRNA-seq) alongside multi-omics platforms (including spatial transcriptomics, proteomics, and metabolomics) has begun to unravel the cellular trajectories governing treatment response and resistance. These high-dimensional technologies allow researchers to track individual immune subsets-such as the shift in tumor-infiltrating immune cells from exhausted CD8^+^ T cells to progenitor-exhausted phenotypes, or the repolarization of immunosuppressive myeloid populations-directly within their spatial architectures post-oncolysis. By accurately mapping these cellular interactions and metabolic shifts, multi-omics technologies provide a molecular blueprint for predicting patient outcomes and rationally designing the next generation of personalized, OV-based combinatorial regimens (Le et al., 2025).

## 13 Safety considerations and clinical toxicities of oncolytic virotherapy

While OVs show significant therapeutic promise as a novel modality in cancer immunotherapy, their safety profile warrants careful consideration to ensure successful clinical translation. The clinical administration of OVs, whether via intratumoral or intravenous routes, can trigger a spectrum of adverse events that range from mild, predictable immune reactions to severe, systemic toxicities (Rahman and McFadden, 2021).

### 13.1 Inflammatory toxicities and cytokine release syndrome

The most common adverse effects observed following OV administration are acute inflammatory toxicities. Patients frequently experience constitutional flu-like symptoms, including transient fever, chills, fatigue, and myalgia, which are typically self-limiting and manageable with standard antipyretics. These symptoms are secondary to the immediate innate immune recognition of viral pathogen-associated molecular patterns (PAMPs) (Cook and Chauhan, 2020).

However, in cases of robust viral replication or high-dose systemic delivery, escalated immune activation can lead to hyperinflammation. This uncontrolled immune response can culminate in cytokine release syndrome (CRS), characterized by a massive, systemic elevation of pro-inflammatory cytokines such as IL-6, IFN-gamma, and TNF-alpha. Severe CRS can result in hemodynamic instability, capillary leak syndrome, and multi-organ dysfunction, requiring intensive monitoring and intervention with corticosteroids or targeted anti-IL-6 therapies (e.g., tocilizumab) (Fan et al., 2026a).

### 13.2 Off-target viral replication and risks in immunocompromised populations

Beyond generalized inflammation, a critical safety consideration unique to replicating viral therapies is the risk of off-tumor viral replication and subsequent off-target infection. Although modern OVs are engineered or naturally selected for tumor-selectivity (exploiting aberrant signaling pathways or defective antiviral machinery in cancer cells), inadvertent targeting or shedding into healthy, non-cancerous tissues remains a potential hazard (Jadhav et al., 2025).

This risk of off-tumor infection is profoundly elevated in immunocompromised patient populations, such as those with advanced malignancies, concurrent lymphopenia, or those undergoing intensive chemotherapy and immunosuppressive regimens. In these patients, impaired immune surveillance and compromised neutralising antibody responses may fail to clear the virus from normal tissues. Consequently, unchecked viral propagation in healthy organs can lead to severe complications, including tissue necrosis, hepatitis, or encephalitis, depending on the viral tropism (Dropulic and Lederman, 2016).

### 13.3 Mitigation strategies

To mitigate these clinical risks, future advancements in OV therapy must prioritize the optimization of stringent patient selection criteria, particularly evaluating the immune status of candidates. Additionally, incorporating safety switches (e.g., inducible suicide genes) into the viral genome and establishing standardized toxicity management protocols are imperative strategies to balance anti-tumor efficacy with patient safety (Bi et al., 2026).

## 14 Tumor-specific customization of OV-chemotherapy combinations

It is critical to recognize that the therapeutic efficacy of OV and chemotherapy combinations is highly dependent on the distinct histopathological and immunological profiles of different tumor types. A generalized framework that treats all malignancies uniformly overlooks the complex physiological barriers and immune landscapes that dictate viral permissivity, intratumoral dissemination, and subsequent therapeutic synergy. Therefore, optimizing combination regimens requires strict customization tailored to the unique microenvironmental features of specific cancers (Garmaroudi et al., 2022).

### 14.1 Overcoming physical barriers in stroma-dense tumors: the case of pancreatic cancer

Pancreatic ductal adenocarcinoma (PDAC) represents a classic archetype of a highly restrictive, “cold” TME. PDAC is characterized by a dense, desmoplastic stroma composed of extracellular matrix (ECM) proteins, activated pancreatic stellate cells, and a high interstitial fluid pressure. This dense stroma creates a formidable physical barrier that severely hinders intratumoral virus spread, restricts the penetration of co-administered chemotherapeutic agents, and limits the infiltration of CTLs (Arnold et al., 2026).

To overcome these structural impediments, OV-chemotherapy strategies in stroma-dense tumors must be dynamically customized. Rather than relying solely on standard cytotoxic drugs, combinations should incorporate agents capable of remodeling or depleting the stroma. For instance, combining OVs with chemotherapies that target cancer-associated fibroblasts (CAFs) or co-administering matrix-degrading enzymes (such as hyaluronidase) can effectively disrupt the stromal architecture. This structural debulking enhances the fluidics of the TME, thereby facilitating both viral dissemination throughout the tumor mass and deeper penetration of small-molecule chemotherapeutic agents (Xiao et al., 2026).

### 14.2 Exploiting T-cell inflamed microenvironments: the case of melanoma

In stark contrast to pancreatic cancer, malignant melanoma frequently presents a highly T-cell inflamed, “hot” tumor microenvironment. Melanoma typically exhibits a high tumor mutational burden (TMB) and a rich baseline infiltration of tumor-infiltrating lymphocytes (TILs), making it inherently more immune-permissive and responsive to immunotherapeutic interventions (Khosravi et al., 2024).

In this context, the primary objective of OV-chemotherapy combinations shifts from overcoming physical barriers to amplifying ICD and reversing local immunosuppression (such as down-regulated MHC molecules or active checkpoint pathways). For melanoma, OVs are ideally paired with immunomodulatory chemotherapies (e.g., low-dose cyclophosphamide to deplete regulatory T cells, or dacarbazine) that synergistically augment viral-mediated immunogenicity. This combination drives a robust abscopal effect and primes the TME, making it exceptionally amenable to sequential or concurrent immune checkpoint inhibition (such as anti-PD-1 or anti-CTLA-4 therapies) (Li et al., 2025b; Xiao et al., 2026).

### 14.3 Framework for tailored regimens

Ultimately, a “one-size-fits-all” approach to OV-chemotherapy design is insufficient to achieve optimal clinical outcomes. Future clinical trial designs must stratify patients based on tumor-specific parameters, including stroma density, vascularization, and baseline immune infiltration status. Customizing the selection and timing of the chemotherapeutic partner based on these parameters represents the most promising avenue for maximizing the synergistic potential of oncolytic virotherapy (Liu et al., 2025a; Tiwari et al., 2026).

## 15 Oncolytic virotherapy: from reversing immune tolerance to clinical translation and future frontiers

Oncolytic virotherapy represents a potent strategy to dismantle established immune tolerance by fundamentally reprogramming the immunosuppressive TME. Beyond direct oncolysis, viral infection induces a profound shift in localized cytokine profiles, converting the TME from a tolerogenic “cold” environment into a pro-inflammatory “hot” landscape. This inflammatory milieu significantly blunts the suppressive capacity of Tregs and MDSCs, while concurrently driving the M1-like antitumor polarization of TAMs. Furthermore, OV-mediated inflammation upregulates the machinery required for antigen presentation and robust T-cell infiltration, effectively reversing tumor-evoked immune evasion and fostering systemic, durable antitumor immunity (Zhang et al., 2021).

Driven by these precise immunomodulatory and microenvironmental mechanisms, the clinical landscape of oncolytic virotherapy has expanded drastically over the past decade. A diverse array of viral backbones, including talimogene laherparepvec (T-VEC), adenoviruses, reoviruses, and vaccinia viruses, have successfully advanced through various phases of clinical evaluation. Reflecting our deeper understanding of the TME, modern clinical paradigms have shifted decisively away from monotherapies toward rational combination strategies. The integration of OVs with conventional chemotherapies, immune checkpoint inhibitors (e.g., anti-PD-1/PD-L1), and targeted molecular therapies now represents the vanguard of current oncology trials, yielding highly encouraging outcomes and enhanced objective response rates across a broad spectrum of advanced solid tumors (Wu et al., 2023).

Despite these promising translational milestones, several formidable hurdles restrict the widespread, uniform efficacy of OVs in routine clinical practice. Chief among these translational challenges are optimizing systemic delivery methods to bypass intravascular neutralization, overcoming pre-existing or treatment-induced neutralizing antiviral immunity, and delicately balancing the destructive host antiviral response with the constructive antitumor immune response. Addressing these multi-layered constraints defines the future directions of the field. Overcoming these barriers will necessitate next-generation genetic engineering strategies-such as chemical shielding of viral capsids or arming OVs with immunomodulatory transgenes-alongside the rational design of mechanistic combination therapies and the discovery of robust, biomarker-driven patient stratification strategies to achieve the full potential of personalized oncolytic medicine (Wu et al., 2023).

## 16 Mechanistic interplay and synergies between oncolytic viruses and chemotherapy

To amplify the reversal of immune tolerance within recalcitrant tumors, the strategic combination of OVs with traditional chemotherapeutic regimens has emerged as a major clinical frontier. As synthesized by Wu et al. (2023), select chemotherapeutic agents function as potent immunomodulators capable of priming the TME for enhanced viral and immune action. This priming is achieved primarily through the induction of ICD, a process that drives the spatiotemporal release of damage-associated molecular patterns (DAMPs) and tumor antigens, while concurrently depleting immunosuppressive cell populations to lower the threshold for effective host immune activation.

When co-administered, chemotherapy and OVs exhibit a multifaceted, reciprocal synergy that addresses both physical and immunological barriers. Mechanistically, chemotherapeutic disruption of the dense tumor stroma alleviates physical constraints, thereby enhancing intratumoral viral dissemination and spread. Simultaneously, the transient suppression of antiviral immunity by chemotherapy permits robust initial viral replication. Reciprocally, OVs function as powerful chemosensitizers; by altering cell-cycle regulation, lowering the threshold for apoptosis, and amplifying DNA damage responses, OVs render otherwise resistant tumor cells highly susceptible to cytotoxic agents (Wu et al., 2023).

This powerful bidirectional synergy is strongly supported by accumulating preclinical evidence, which consistently demonstrates that combinatory OV-chemotherapy regimens significantly improve tumor regression and prolong overall survival compared to either monotherapy (Wu et al., 2023). Translating these insights into clinical paradigms, regulatory-approved platforms such as T-VEC and other emerging viral backbones have exhibited heightened therapeutic efficacy, superior tumor control, and improved objective response rates when integrated into systemic combination frameworks with conventional cytotoxic drugs.

## 17 Cellular and infection microbiology of oncolytic virotherapy

### 17.1 Oncolytic platforms as engineered pathogens and host PRR sensing axis

From a cellular and infection microbiology perspective, the therapeutic efficacy of virotherapy relies on utilizing OVs as sophisticated, engineered infectious agents. These platforms are meticulously designed to exploit cell-autonomous defects in antiviral machinery-such as aberrant IFN signaling pathways-that are inherent to neoplastic cells. Current genetic engineering strategies focus on the precise deletion of viral virulence genes and the insertion of tumor-specific promoters, maximizing selective replication within malignant tissues while ensuring a stringent clinical safety profile (Wu et al., 2023). A comprehensive summary of representative oncolytic viral platforms, including their unique microbiological features and genetic modifications, is provided in **Table 1**.

**Table 1:** Representative oncolytic viruses and their key features from an infection microbiology perspective.

Oncolytic virus	Viral family	Genome type	Key infection microbiology features	Immunological effects	Clinical status
Herpes simplex virus-1 (T-VEC)	Herpesviridae	dsDNA	Engineered deletion of neurovirulence genes; efficient cytoplasmic–nuclear trafficking	GM-CSF–mediated DC activation; enhanced T cell priming	FDA-approved (melanoma)
Adenovirus	Adenoviridae	dsDNA	Tumor-selective replication via defective p53/Rb pathways	Strong innate sensing; promotes adaptive immunity	Phase I-III
Reovirus	Reoviridae	dsRNA	Preferential replication in Ras-activated cells; TLR3 activation	Potent NK cell and IFN responses	Phase I-II
Vaccinia virus	Poxviridae	dsDNA	Broad tropism; cytoplasmic replication; large transgene capacity	Robust innate and adaptive immune activation	Phase I-II
Measles virus	Paramyxoviridae	ssRNA (-)	CD46/SLAM receptor usage; syncytia formation	Strong immunogenic cell death	Phase I

The introduction of these engineered pathogens into the TME triggers a complex cascade of host-pathogen interactions mediated by PRRs. Both tumor cells and infiltrating immune cells express specialized sensors, most notably TLR3, to detect viral dsRNA intermediates generated during replication. Ligand binding to TLR3 initiates a downstream signaling cascade governed by the toll/IL-1 receptor domain-containing adapter inducing IFN-beta (TRIF) dependent pathway. This axis culminates in the robust activation of key transcription factors, including IRF3 and NF-kappaB, which drive the localized production of type I interferons and pro-inflammatory chemokines (Wu et al., 2023). The specific intracellular molecular pathways and their downstream immunological outcomes are detailed in **Table 2**.

**Table 2:** Innate immune sensing pathways activated by oncolytic viruses in the tumor microenvironment.

Sensor pathway	Ligand	Major signaling molecules	Key immune outcomes	Relevance to immune tolerance
TLR3	Viral dsRNA	TRIF, IRF3, NF-κB	Type I IFN, pro-inflammatory cytokines	Breaks tumor-induced immune suppression
cGAS-STING	Cytosolic DNA	STING, TBK1, IRF3	IFN-β, DC activation	Enhances antigen presentation
RIG-I/MDA5	Viral RNA	MAVS, IRF3/7	Antiviral and inflammatory responses	Converts “cold” tumors to “hot”

### 17.2 Innate immune cascades: natural killer cells and macrophage reprogramming

Beyond direct intracellular sensing, OVs orchestrate a profound restructuring of innate immune cell networks within the TME, with NK cells serving as critical first-responders. OV infection acutely accelerates the recruitment of NK cells to the tumor core, enhances their intrinsic cytotoxicity against both virus-infected and uninfected neighboring malignant cells, and triggers robust production of IFN-gamma (Wu et al., 2023). These multifaceted, context-dependent roles of NK cells during the early kinetics of virotherapy are categorized in **Table 3**.

**Table 3:** Roles of innate immune cells during oncolytic virus infection in cancer.

Immune cell type	Infection microbiology role	Anti-tumor function	Therapeutic implication
Natural killer cells	Early antiviral responders; kill infected cells	Direct cytotoxicity; IFN-γ production	Timing of NK activation is critical for OV efficacy
Macrophages/TAMs	Viral sensing and clearance	M2-to-M1 reprogramming; antigen presentation	Key mediators of immune tolerance reversal
Dendritic cells	Antigen uptake from infected cells	T cell priming and memory formation	Central to systemic anti-tumor immunity

Parallel to NK cell activation, macrophage plasticity represents another pivotal regulatory axis manipulated by OVs. TAMs, which typically adopt a pro-tumorigenic and immunosuppressive M2-like phenotype, undergo profound phenotypic reprogramming upon exposure to OV-induced inflammatory cascades. OVs successfully subvert this tolerogenic state, driving TAMs toward a classically activated, pro-inflammatory M1-like state (Wu et al., 2023). This phenotypic switch significantly enhances the cross-presentation of tumor-associated antigens and coordinates downstream adaptive immunity, as illustrated in the interaction networks in **Table 3**.

## 18 Conclusion

The programmatic reversing of the cold, immunosuppressive tumor microenvironment represents a fundamental prerequisite for achieving sustained clinical responses in oncolytic virotherapy. Rather than relying on a singular mechanism of action, modern therapeutic paradigms increasingly emphasize a multi-synergistic framework that simultaneously exploits viral-mediated oncolysis, innate immune pattern recognition receptor activation, and the subversion of local immune checkpoint networks. As highlighted by Bayode et al. (2024), modulating these overlapping axes allows OVs to serve as dynamic biological platforms that dismantle the dominant immunosuppressive architecture of the stroma. This multi-synergistic disruption not only remodels the local cytokine milieu but also successfully drives the systemic recruitment and expansion of tumor-reactive cytotoxic T lymphocytes, providing a robust rationale for combining OVs with traditional cytotoxic or immunomodulatory regimens (Bayode et al., 2024).

OV-chemotherapy combinations are a powerful, mechanism-driven strategy in cancer immunotherapy. The therapeutic success of this combinatorial approach relies fundamentally on the precise temporal orchestration among viral replication, the induction of ICD, and chemotherapy-driven myeloid modulation. TLR-mediated innate immune tuning to maximize adaptive priming. Biomarker-informed personalization to exploit the immunogenic window while avoiding premature immune suppression.

Future studies integrating OVs biology, chemotherapy pharmacodynamics, and immune monitoring are poised to optimize these combinations, potentially converting non-inflamed tumors into highly immunogenic, therapy-responsive lesions. OVs represent a unique therapeutic platform capable of directly killing tumor cells while simultaneously overcoming immune tolerance and enhancing anti-tumor immunity. Their integration with chemotherapy offers synergistic benefits that address both tumor-intrinsic and immune-mediated mechanisms of resistance (**Table 4**). Continued advances in immunology, virology, and clinical trial design are expected to further establish oncolytic virotherapy as a key component of multimodal cancer treatment.

**Table 4:** Synergistic mechanisms between oncolytic viruses and chemotherapy.

Chemotherapy effect	Impact on OV infection	Immunological consequence	Therapeutic benefit
Immunogenic cell death	Enhanced antigen release	Increased T cell priming	Improved tumor control
Transient immunosuppression	Reduced antiviral clearance	Increased viral replication	Enhanced oncolysis
Stromal disruption	Improved viral spread	Greater immune cell infiltration	Synergy with immunotherapy
